# Research on Adaptive Management of the Social–Ecological System of a Typical Mine–Agriculture–Urban Compound Area in North Shanxi, China

**DOI:** 10.3390/ijerph19148681

**Published:** 2022-07-17

**Authors:** Shufei Wang, Shengpeng Li, Kai Yang, Yi Feng, Shihan Liu, Jianjun Zhang, Yingui Cao, Zhongke Bai

**Affiliations:** 1 School of Land Science and Technology, China University of Geosciences, Beijing 100083, China; wangshufei@cugb.edu.cn (S.W.); 3012210008@cugb.edu.cn (S.L.); 2112200006@cugb.edu.cn (K.Y.); 2012200028@cugb.edu.cn (Y.F.); liushihan@cugb.edu.cn (S.L.); baizk@cugb.edu.cn (Z.B.); 2 Key Laboratory of Land Consolidation and Rehabilitation, Ministry of Natural Resources, Beijing 100035, China

**Keywords:** adaptive management, sustainable development, social–ecological system, mine–agriculture–urban compound area

## Abstract

The mine–agriculture–urban compound area formed under the combined effects of natural conditions, mineral resource endowments, and historical development is affected by severe man-made disturbances, and faces a prominent contradiction between economic development and ecological protection. Guiding the future development is an urgent problem in this region. This research used image data, logical reasoning, and empirical analysis, based on social and economic statistics and land-use data, to analyze the typical characteristics and problems of the social–ecological system in the mine–agriculture–urban compound area. Moreover, we identified future directions for the region guided by policy documents and built a philosophy framework for sustainable development and management of the region based on the concept of adaptability. The results showed the following: (1) At present, the output value of the coal industry accounts for 84.10% of the total regional output value, severely disturbing its social–ecological system, which needs to be protected and restored under human guidance and management. (2) The future development of this region depends on the one hand on green mining, and on the other hand, it is necessary to fully tap the potential of arable land and livestock farms to develop efficient and intensive agriculture. (3) The key contents of the social–ecological system management of the mine–agriculture–urban compound area include resolving the contradiction between development and protection, ensuring development, optimizing industrial structure, and safeguarding public interests. In conclusion, this research can expand the connotation and application scope of adaptive management and provide a reference for such areas facing the prominent contradiction between development and protection.

## 1. Introduction

From the perspective of world development and energy history, energy is one of the main drivers of human economic and social development [1]. Coal resources are the most important primary energy sources [2]. The regions supplying coal resources provide material, capital, talents, and employment opportunities, and promote industrialization and national economic and social development [3]. However, many problems accompany the development of coal resource mining areas, including the overweight socio-economic structure formed by rapid development and the ecological problems caused by coal resource mining [4,5]. With the continuous improvement of scientific cognition and technology, we have come to realize that natural ecological systems and human social systems are intertwined to form a complex social–ecological system [6,7]. This system is a nonlinear, dynamically coupled system formed by the interaction of one or more social and ecological subsystems [8]. It is an adaptive system that is closely linked with nature, and under the influence of its own disturbances and force as well as external ones, it forms characteristics such as unpredictability, self-organization, a multi-stable state, the threshold effect, history dependence, etc. [9,10]. The mutual feedback mechanism is its core content [11].

The mine–agriculture–urban compound area is a special type of area formed in the process of resource exploitation and regional development. In 2015, Cao firstly proposed and defined the “mine–agriculture–urban compound area” as a community of resources, economy, and society in which exist the material circulation and energy flow of the development process, where mineral resource exploitation, processing and service, agricultural production, and biological resources utilization are the primary industries [12]. The development of this region involves economy, society, and ecology. Under the dual goals of economic and social development and ecological and environmental protection, a complex social–ecological system is formed in this region. An analysis of the above connotations indicates that the social–ecological system of the mine–agriculture–urban compound area refers to a system in which the human economy and society and the natural ecological environment are closely linked, interdependent and coupled under the joint action of mineral resource mining and processing, agricultural production, and urban development [12]. Especially in China, on the one hand, coal resource-rich areas are mostly coupled with areas having a fragile ecological environment [13]; on the other hand, large-scale coal bases are mainly distributed in arid or semi-arid areas at this stage [14]. That is, the social–ecological system in China’s mine–agriculture–urban compound area is faced with a prominent contradiction between ecological environment protection and economic and social development, and the future development of this region will be subject to severe challenges. Furthermore, in view of the above-mentioned characteristics and problems of the social–ecological system in this compound area, adaptive management has become a guiding concept [11,15,16].

With the exploitation of resources and the construction of mining areas, China has formed a mining administrative unit with complete political, economic, and cultural systems [12]. The difference is that the function of forming mining areas abroad is more specific for resource supply [12]. In the process of managing mining areas abroad, greater attention is paid to the harmonious relationship and development at the social level [17]. Among these mining areas, the use of adaptive management to guide land use is prevalent [18,19]. Through in-depth research on the development needs and stakeholder choices at the social level, based on adaptive management, future land-use goals and methods that can coordinate ecology, economy, and society are proposed [17,18]. In addition, adaptive management is applied to the process of ecological environment governance in mining areas at home and abroad. For example, the restoration and reuse of abandoned mining areas can be achieved by planting regional adaptive tree species and implementing adaptive management monitoring [18,19,20].

Driven by scholars such as C.S. Holling and Kai N. Lee, adaptive management was proposed as a way to overcome the limitations of static evaluation and environmental management and as an effective method and experiment to cope with uncertainty [21,22]. Subsequently, many scholars have understood adaptive management to be a goal-oriented or hypothesis-oriented continuous, systematic, learning-by-doing process, which includes basic planning, monitoring, research, and regulation [22,23,24]. With continuous management progress, people’s needs are increasingly valued, and the public and society are integrated into this process [25,26,27,28]. At the same time, the goal orientation is clearer, requiring clear expectations and outputs in management practice [29]. Understanding of the management mechanism has also undergone a process of cyclical framework–spiral process–cyclic ascending action [29,30,31]. In conclusion, in the face of an uncertain future environment, “adaptive management” can be understood as a process whereby, to achieve management objectives and maintain sustainable development [26], managers fully consider the influence factors and stakeholder needs according to the experience, present and possible future situation to continuously investigate, design, plan, implement, monitor, evaluate, and adjust management activities, so as to generate continuous feedback on the implementation of the plan and adjust the management process [32,33]; that is, “adaptive management” is a process of learning from practice to then guide practice [23,31,34]. In this process, the key contents are: (1) Adaptive management is a dynamic management mode in which objectives and means can be adjusted according to the changes in external driving factors. (2) The application of an adaptive management mode can adjust and respond timely when facing the randomness, complexity, and uncertainty of external driving factors. (3) To some extent, the implementation of adaptive management depends on the accuracy of early prediction, the ability to adapt promptly to change, and the degree of tolerance.

With the continuous progress in production and information technology, the development of local society is accompanied by corresponding problems. Social policy is formed by the government’s measures to solve specific social problems. Although social policies are universal and fair, the cognitive and emotional differences of different individuals or groups can lead to different responses to policy implementation, including negative responses. For example, China’s social security policy aims to protect the basic needs of all residents after they are unable to perform productive labor. However, for the vast number of migrant workers who do not have regular jobs, this policy does not benefit their lives, so the phenomenon of migrant workers withdrawing from insurance frequently occurs. As a result, social security, an inclusive policy, is not generally recognized [35]. Therefore, in the process of formulating, implementing, and managing regional development policies, basic investigative work is key, and it is necessary to understand the cognitive and emotional contexts of the target group, obtain the support and participation of individuals or social groups, and finally realize the effectiveness and sustainability of policy and management activities [36].

In summary, this paper proposes the future development goals and management framework of the social–ecological system and its adaptive management in the mine–agriculture–urban compound area based on inheriting the connotation and typical research area of the existing mine–agriculture–urban compound area, sorting out the characteristics of its social–ecological system evolution process and the problems it faces, and adopting the concept of adaptive management. On the one hand, this paper points out the direction for the future development of a typical mine–agriculture–urban compound area against the background of ecological civilization construction. On the other hand, it expands the scope of practical application of the adaptive concept.

## 2. Materials and Methods

### 2.1. Study Area

On the one hand, as mentioned above, in this mine–agriculture–urban compound area, resource exploitation, agricultural production, and economic and social development are closely linked to form a complex adaptive system. Under the influence of external drivers such as national energy policies and local development policies, as well as the internal drivers of the ecosystem, this area exhibits the obvious characteristics of a social–ecological system. Therefore, this region was defined as a typical social–ecological system in the context of increasingly uncertain times. The research on it will further enrich the social–ecological system research system. On the other hand, the mine–agriculture–urban compound area was developed by relying on China’s first Sino-foreign joint venture coal mining enterprise, which has a research foundation at home and abroad. This area is located in the unique Loess Plateau in China, which is an area representative of the contradiction between resource exploitation and environmental protection. Therefore, selecting it as the case study had high reference significance and promotion value for China and the world. In summary, this study uses the mine–agriculture–urban compound area as a case study from the perspective of particularity and representativeness.

**Figure 1:**
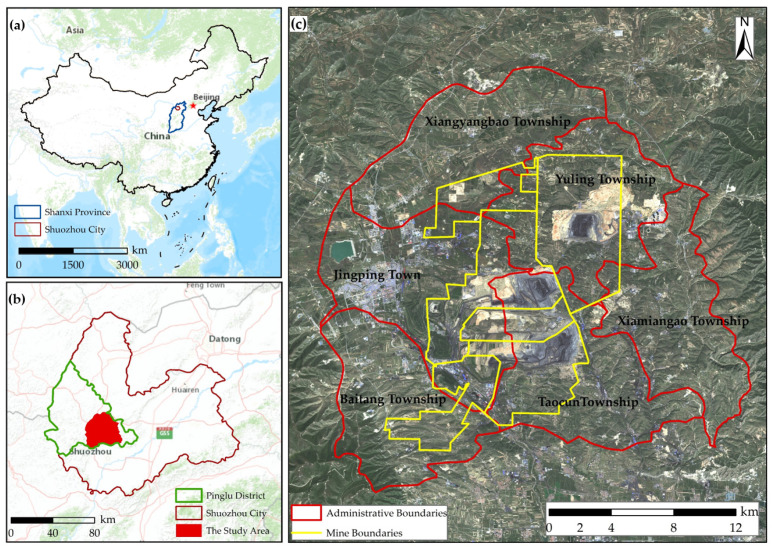
The location of the mine–agriculture–urban compound area. (The base maps of (**a**,**b**) are the color version of the map of China that comes with ArcGIS, the base map of (**c**) is the 2020 Landsat remote sensing image data that was from *United States Geological Survey* (https://earthexplorer.usgs.gov/ accessed on 3 March 2022), and the figure was made by Shufei Wang).

The above-mentioned mine–agriculture–urban compound area is located in Pinglu District, Shuozhou City, Shanxi Province of China (39°21′–39°58′ N, 111°52′–112°41′ E) (Figure 1), which is located in the Loess Plateau hills of north Shanxi Province, where the topography is higher in the northwest than southeast [12,37]. This area is in the northern temperate semi-arid continental monsoon climate zone, where the climate is cold, dry, windy, and sandy with an average annual temperature of 4.50 °C [12]. The zonal soil type is loessial soil and chestnut soil, and the zonal vegetation type is steppe vegetation [12,37,38]. There are many mountains and deep ditches, and there is minimal flat land, serious soil erosion and water loss, low vegetation coverage, and a fragile ecosystem background [12]. The agriculture comprises a diversified agricultural product system represented by its famous wheat (*Triticum aestivum L.*) [12]. The Pinglu District is a typical northern resource-based town, where there are more than 40 kinds of proven mineral resources, mainly coal resources. On the one hand, the coal mines in the study area are the largest and most profitable in Shuozhou, which has made Shuozhou a growing coal resource-based city. On the other hand, according to the *Territorial Spatial Planning of Shuozhou City (2020–2035)*, the study area is the ecological development center for the Shuozhou City and serves the purpose of realizing ecological civilization. Thus, attaching great importance to, developing, and managing this research area are important to ensuring the economic development and ecology of Shuozhou City [39].

**Figure 2:**
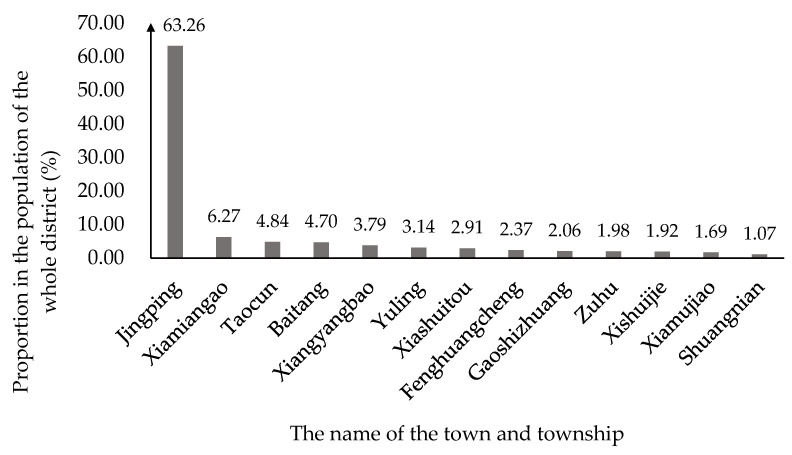
The population sizes of townships in Pinglu in 2020. (The data were compiled from the *Shanxi Statistical Yearbook* in 2020, and the figure was made by Shufei Wang).

According to Cao, this mine–agriculture–urban compound area includes Jingping Town, Xiamiangao Township, Taocun Township, Baitang Township, Xiangyangbao Township, and Yuling Township [12]. According to the *Seventh National Census Bulletin of Pinglu*, the total resident population in 2020 was approximately 1.48 × 10^5^, among which the mine–agriculture–urban compound area accounted for 86.00% of the total population and was the main area of human economic and social activities (Figure 2). According to the *Integrated Land Use in Pinglu of Shuozhou City (2006–2020)*, the cultivated land area of the mine–agriculture–urban compound area accounted for 30.38% of the cultivated land area of Pinglu. Meanwhile, the main, large-scale coal mining area in Pinglu is the China Coal Pingshuo Mining District, which is in this mine–agriculture–urban compound area and contains three large opencast mines, namely, Antaibao, Anjialing, and East Opencast, and three large modern underground mines, I, II, and III. Moreoer, the China Coal Pingshuo Mining District is the largest and most modern mining area of the 100 million tons of opencast and underground mining in China, and is one of the 13 coal bases in China [40]. Therefore, the mine–agriculture–urban compound area can represent the situation of Pinglu District in terms of economic and social development and ecological environment governance.

### 2.2. Data Source and Processing

**Figure 3:**
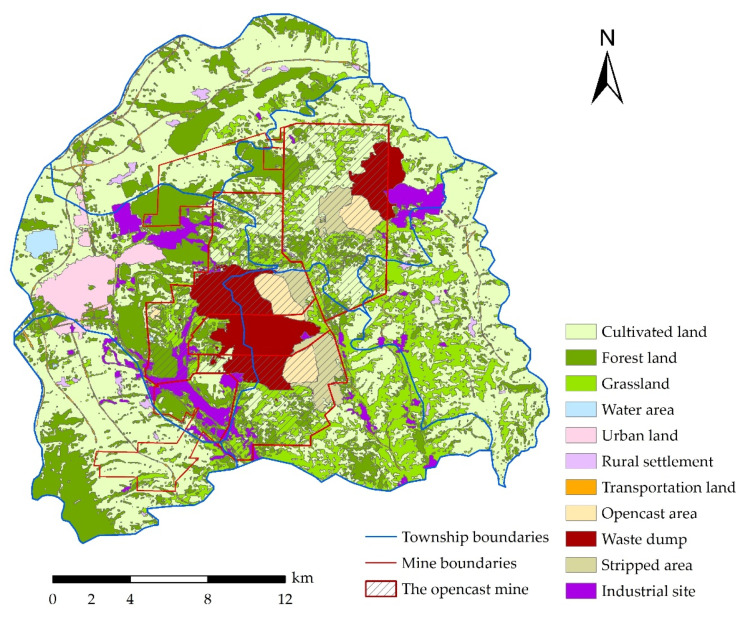
The status of land use in the typical mine–agriculture–urban compound area. (The Landsat TM remote sensing image was from *United States Geological Survey* (https://earthexplorer.usgs.gov/ accessed on 3 March 2022), and the figure was made by Shufei Wang).

The remote sensing image was downloaded from the official website of the *United States Geological Survey* (USGS) (https://earthexplorer.usgs.gov/ accessed on 18 December 2021). In this study, the Landsat TM remote sensing image in 2019 was selected with a resolution of 30 m. After systematic radiometric correction and geometric correction, software such as ENVI 5.2 (Exelis Visual Information Solutions, New York, NY, America) and ArcGIS 10.2 (Environmental Systems Research Institute, Redlands, CA, America) were used to select the required bands for fusion, mosaic, and cropping. In view of the characteristics of highly complex land-use types, fragmented landscapes, and complex terrain in the mine–agriculture–urban study area, the 5-4-3 standard false color band was used to supervise and classify the land-use types in the study area. The classification standard was mainly based on the *Classification Standard of Land Use Status (GB/T 21010-2017)* and combined with the actual land-use status of the study area. The land-use types were divided into 11 categories, including cultivated land, forest land, grassland, urban land, rural settlement, transportation land, opencast area, waste dump, stripping area, industrial site, and water area. We manually corrected the misclassification and omission in the interpretation results, referring to Google Earth to maximize the classification accuracy. The classification results are shown in Figure 3. The land-use data were used to analyze the characteristics of land use and ecological environment in the study area, and to serve as one of the bases for determining future development and management goals in the study area.

The social and economic statistics were arranged from the *Shanxi Statistical Yearbook*, which were used to analyze the economic and social development level of the study area, and to serve as one of the bases for determining future development and management goals in the study area. The regional development data and situation introduction were arranged from the *Pinglu County Chronicle*, which were used to summarize the development history and laws of the study area. The policy documents were arranged from the website of *The People’s Government of Shanxi Province* (http://www.shanxi.gov.cn/ accessed on 18 December 2021), which were used to determine the future development and management goals of the study area. All the above documents were officially released by the government and were reliable.

In addition, we initially identified the main social problems in the study area through random face-to-face interviews with residents of the study area. The interviewees included farmers, grass-roots managers, freelancers, and working and retired employees of coal mining enterprises. The interviews mainly concerned income differences, life satisfaction, policy satisfaction, and government satisfaction. Ultimately, we obtained 52 valid interview results.

### 2.3. Research Process

Firstly, we summarized the development history of Pinglu District or the mine–agriculture–urban compound area and identified its development trend and development goals.

Secondly, we analyzed the status of the regional ecosystem based on the land-use status map, analyzed the regional socio-economic status based on statistical data and the results of face-to-face interview survey, and combined the two aspects to analyze the status of the regional social–ecological system.

Thirdly, we proposed the future development direction and its management framework for the region combined with the regional development trends, goals, the status of the social–ecological system, and the guidance of the concept of adaptive management.

Finally, we compared the development goals and management framework with future development plans of the research area to verify the applicability of the results of this research.

It should be noted that, because of the limited availability of data, the social and economic statistics data were from Pinglu District, and the land-use data were from the mine–agriculture–urban compound area. However, as mentioned above, the mine–agriculture–urban compound area can well represent the situation of Pinglu District. Therefore, data acquisition and analysis were feasible.

## 3. Results

### 3.1. The Development Process

**Figure 4:**
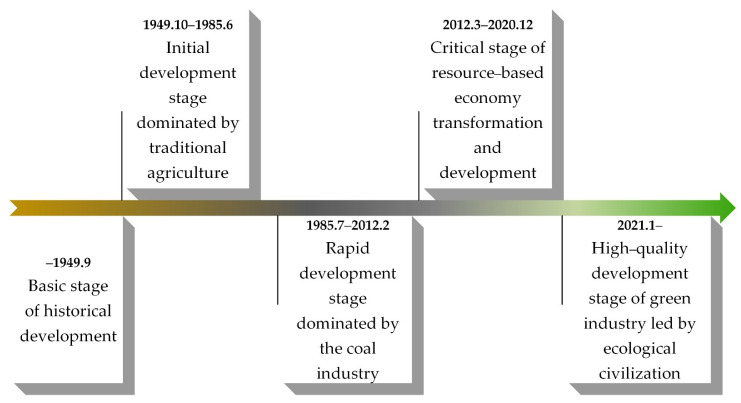
The development process of Pinglu. (The line with arrow represents the time sequence of Pinglu’s development; the yellow line represents the historical development foundation stage and the initial development stage relying on the traditional characteristic industries in the loess area; the black line represents the rapid development stage and part of the transformation critical stage mainly relying on coal; and the green line represents part of the transformation critical stage and the high-quality development stage seeking the transformation of resource-based towns and the green development road. The data were compiled from the policy documents published by the *People’s Government of Shanxi Province* (http://www.shanxi.gov.cn/ accessed on 18 December 2021), and the figure was made by Shufei Wang).

At the beginning of the economic reform and open up in China, Pinglu’s economic level was the last in Shanxi Province and the second-to-last in China with per capita income of only CNY 20.10 (USD 13.43) [41], which was in the “initial development stage dominated by traditional agriculture”. Under the guidance of the economic reform and opening up, Pinglu gave full play to its resource advantages to develop the coal economy and entered the “rapid development stage dominated by the coal industry” [12]. During this period, Pinglu began to manage and rebuild the ecological environment damaged by mining since 1990 [42,43] and entered the “exploration stage of balanced development of economy, society and ecology” to establish the precedent of ecological restoration of Chinese mines. In 2012, the coal price began to drop, causing Pinglu, Shanxi Province, and even the coal resource-based towns across the country to face huge economic downward pressure. Against the background of the strategic decision regarding “ecology civilization construction” proposed at the 17th CPC National Congress in 2007 and the strategy of “rejuvenating the province through ecology” proposed by Shanxi Province in 2009, and under the guidance of the *Action Plan for Implementing the Opinions of The State Council to Support Shanxi Province to Further Deepen Reform and Promote the Transformation and Development of Resource-based Economy*, Pinglu actively explored the transformation and upgraded development mode and entered the “critical stage of resource-based economy transformation and development” [44,45]. By the end of 2020, Pinglu transformation and reform had achieved phased results, the transformation system and mechanism were basically established, and it entered the “high-quality development stage of green industry led by ecological civilization”, with energy reserve, low-carbon transformation, ecological conservation, happiness, and livability as the future development goals [39]. The summary of the development process of the study area is shown in Figure 4.

The above development process of the study area further indicates that this area is a typical social–ecological system. First, the research area constantly adapts to the development of history and the progress of the times, dynamically adjusts the development direction and plan to exhibit the characteristics of self-adaptation, unpredictability, and self-organization. In the process, this system would continue to inherit the development foundation of the previous period and show the characteristics of historical dependence. Secondly, the reason why the study area constantly adjusts the coal resource development strategy is that the existing development mode is unsustainable, which eventually leads to coal resource exhaustion and regional backwardness. Therefore, it is necessary to reasonably plan and transform the development plan based on considering the threshold effect.

**Figure 5:**
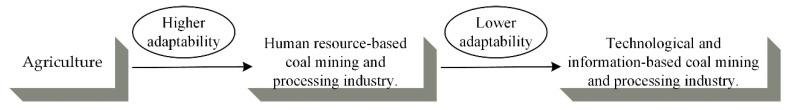
The adaptation of residents in the study area to changes in living or production patterns. (The contents were compiled from Yingui Cao’ doctoral dissertation and the policy documents published by the *People’s Government of Shanxi Province* (http://www.shanxi.gov.cn/ accessed on 18 December 2021), and the figure was made by Shufei Wang).

During the development of the study area, the residents are constantly adapting to the changes in the area (Figure 5). Before the coal mine construction, residents mainly engaged in agricultural production. In the process of coal mine construction, under the leadership of managers and technicians, residents participated in coal mine construction activities, and their income increased. At the same time, this area attracted many foreigners. During this process, although the agricultural production mode was quickly transformed into industrial production, residents showed a positive attitude and high adaptability driven by interests. In the process of coal mining, land needed to be acquired, and the residents who originally lived there needed to be relocated to other places. Four large-scale residents’ relocation activities were carried out in the study area in 1985, 1998, 2008 and 2011 [12]. During this process, residents moved from rural areas to towns, and from independent courtyards to buildings. Their living environment and way of life changed greatly, which takes a long time to adapt to. During this process, most residents showed a positive attitude, because their new living environment was cleaner and more convenient, and at the same time, they could obtain regular financial and material compensation from the coal mining enterprises. With the technology advancement, machines have replaced most of the labor force, so worker layoffs in the coal mining enterprises occurred. Moreover, most laid-off workers were engaged in low-skill jobs, which lead to problems in their re-employment and living security. Among them, some started to work by earning an unstable income in the local labor market, others found work in other cities. During this process, industrial production was quickly transformed to other production methods, but the loss of stable occupations and incomes creates negative attitudes and thus lower adaptability of the residents.

### 3.2. Characterization of Social–Ecological System

**Figure 6:**
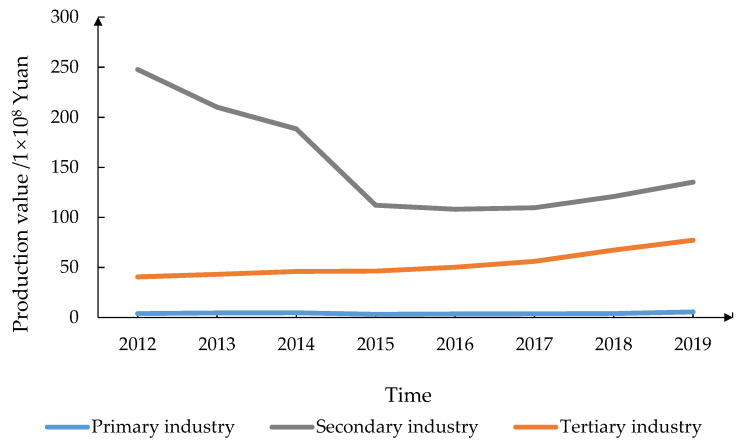
The changes in the industrial structure of Pinglu from 2012 to 2019. (The data were compiled from the *Shanxi Statistical Yearbook* from 2013 to 2020, and this figure shows the changing trend of the proportion of the output value of the three major industries in the total output value, which was made by Shufei Wang).

According to the *Shanxi Statistical Yearbook* from 2013 to 2020, during the period of economic transformation from 2012 to 2019, the resident population of Pinglu increased by 6612, and rural and urban populations accounted for 44.40% and 55.60% in 2019, respectively. The regional GDP in Pinglu decreased by 25.42%. The output value of the primary industry increased from CNY 3.93 × 10^8^ (USD 0.63 × 10^8^) to CNY 5.50 × 10^8^ (USD 0.77 × 10^8^), and the proportion of primary industry increased from 1.34% to 2.52%, mainly involving the planting industry, with the application of agricultural fertilizers at a high level. The output value of the secondary industry decreased from CNY 247.71 × 10^8^ (USD 39.63 × 10^8^) to CNY 135.21 × 10^8^ (USD 18.94 × 10^8^), and the proportion of secondary industry decreased from 84.79% to 62.05%, and industrial assets decreased by 36.57%. The output value of the tertiary industry increased from CNY 40.51 × 10^8^ (USD 6.48 × 10^8^) to CNY 77.19 × 10^8^ (USD 10.81 × 10^8^), and the proportion of tertiary industry increased from 13.87% to 35.42% (Figure 6). Thus, mining is still the dominant industry in Pinglu. According to the data of *Analysis of Economic Operation of Pinglu in the First Half of 2021*, the total production value of coal enterprises accounted for 84.10% of the total production value in Pinglu. That is, the economic and social development of Pinglu relies on coal resources and determines the characteristics of the community of life for resources, economy, and society. It is a complex and typical social–ecological system with multiple ecological and social subsystems interacting.

**Figure 7:**
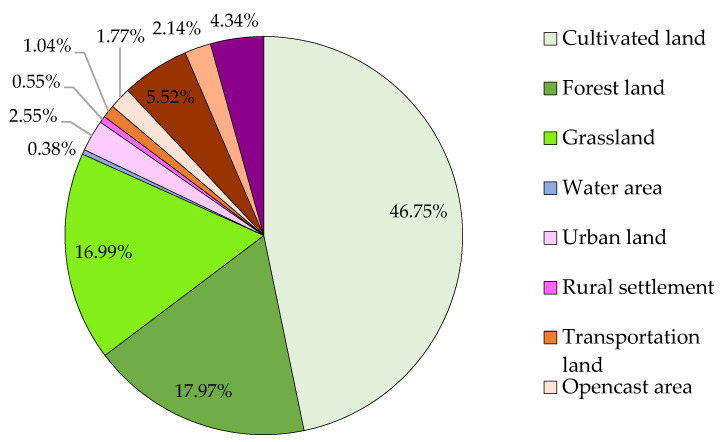
The areal proportion of each land-use type in the typical mine–agriculture–urban compound area. (The data were extracted from Figure 3, and the figure was made by Shufei Wang).

This mine–agriculture–urban compound area is located in the Loess Plateau hills with a fragile ecosystem background [12]. Figure 7 shows that, in this area, cultivated land is the largest component, accounting for 46.75%, but its proportion of the total industry is small (Figure 6). The area of forest and grassland accounts for 34.96%, which supports the development of regional animal husbandry to an extent (Figure 6 and Figure 7). The area of coal mining area accounts for 32.48% (Figure 3), where 13.77% is the mining and dumping area (Table 1). The residential area accounts for 3.10%, the water area accounts for 0.38% and is mainly located in Jingping Town, and transportation land area accounts for 1.04% (Figure 7).

It can be seen that, in this typical mine–agriculture–urban compound area, the severe human disturbance area accounts for at least 65.04%, and there are strong disturbance areas in the ecosystem, such as opencast or underground mining area, dumping area, and restoration area. These strong disturbance areas are located in the middle of the entire mine–agriculture–urban compound area (Figure 3), seriously affecting the landscape connectivity and ecosystem stability of the compound area. At the same time, as mentioned above, the mining boundary area in the study area accounts for 32.48%, that is, the ecosystem directly affected by coal mining in the study area accounts for at least 32.48%. Figure 3 indicates that the affected ecosystems are mainly forest land, grassland, and cultivated land ecosystems. Therefore, there are multiple conflicts among industry, agriculture, urban areas, and ecology in the social–ecological system of the compound area under the disturbances of mining, agriculture, and urban construction based on the fragile background conditions. The main contradictions include the following four aspects: (1) industrial development occupies agricultural land or rural settlements, causing conflicts in compensation standards; (2) the pollutants discharged by industry, agriculture, and urban development seriously endanger the ecological environment, causing conflicts between development and protection; (3) the waste generated by industrial construction affects the residents’ health, causing a conflict between economic benefits and health; (4) rapid urban development and backward rural development leads to the contradiction of unbalanced development. Therefore, the development prospect of this area is complicated and full of uncertainties, and its development process will continue toward a new equilibrium state under the effect of natural, economic, and social laws, forming multiple steady states in different periods. During this period, rational guidance and management by human beings are the key.

In summary, Pinglu relies on the development of the coal industry, which has a significant impact on the regional economic and social development and the ecological environment in the area, and promotes the formation of the existing social–ecological system characteristics. At the same time, combined with the future development goals of the region, it could be seen that the coal industry is still the leading industry for its future development. Therefore, properly handling the relationship between resource utilization, economic and social development, and ecological protection under the industrial structure dominated by the coal industry needs to be considered comprehensively in the future.

### 3.3. The Framework for Adaptive Management

#### 3.3.1. Regional Development Direction and Management Goals

The above analysis indicates that the coal industry is the most important regional characteristic industry in the past, present, and future and basically determines the local economic benefits in Pinglu, but coal mining and processing cause disturbance and damage to the fragile ecological environment. Agriculture is a traditional local industry, with the largest proportion of area, but a low industry proportion. By comparing the agricultural output value per unit area of Shanxi Province and the study area, it was found that the agricultural output value per unit area of the study area (0.78 CNY/m^2^; 0.12 USD/m^2^) was greater than the average value of that of Shanxi Province (0.36 CNY/m^2^; 0.05 USD/m^2^). That is, agriculture is an advantageous industry in the study area. In addition, Figure 3 shows that under the influence of mining activities, the distributions of cultivated land, forest land and grassland in the study area are relatively scattered, which is not conducive to efficient utilization. So, it is necessary to promote the efficient use of regional cultivated land, forest land, and grassland through scientific management decisions, so as to maintain the agricultural advantages of the study area and increase the agricultural output value. Urban development is necessary, but sustainable urban development requires a sustainable economy with a balanced and ecological environment in harmony with it. Realizing this demand requires scientific management decisions.

In addition, in July 2021, the author’s team conducted a face-to-face interview survey in this compound area and found that different interest groups had different needs according to the residents in different occupations and different policy environments. Among them, the compensation and resettlement measures of mining enterprises for different properties varied after land expropriation, which created a sense of disparity among groups and increased social instability factors. People without stable jobs believed that certain policies were not implemented in place, their incomes were not guaranteed, and the degree of public participation in the process of policy formulation and implementation was low. Therefore, improving public participation, strengthening policy interpretation and publicity, and effectively protecting the interests of grassroots people are important guarantees of the stable and sustainable development of the social–ecological system in the compound area.

In summary, this paper argues that the future development direction and management goals of this compound area should proceed from the following four points: (1) through in-depth practice of green ecological development to promote the construction of green mines and properly handle the relationship between resource development and ecological protection; (2) by relying on green mining to continue a sustainable and high-quality energy supply and backup base, and to ensure regional development; (3) to rationally adjust industrial structure, and to economically and intensively use cultivated land, forest, and grassland resources; (4) to attach great importance to the new type of urbanization construction that is ecologically livable and harmonious to achieve a balanced economic, social, and ecological development.

#### 3.3.2. Preliminary Exploration on Adaptive Management Framework of Social–Ecological System of Typical Mine–Agriculture–Urban Compound Area

**Figure 8:**
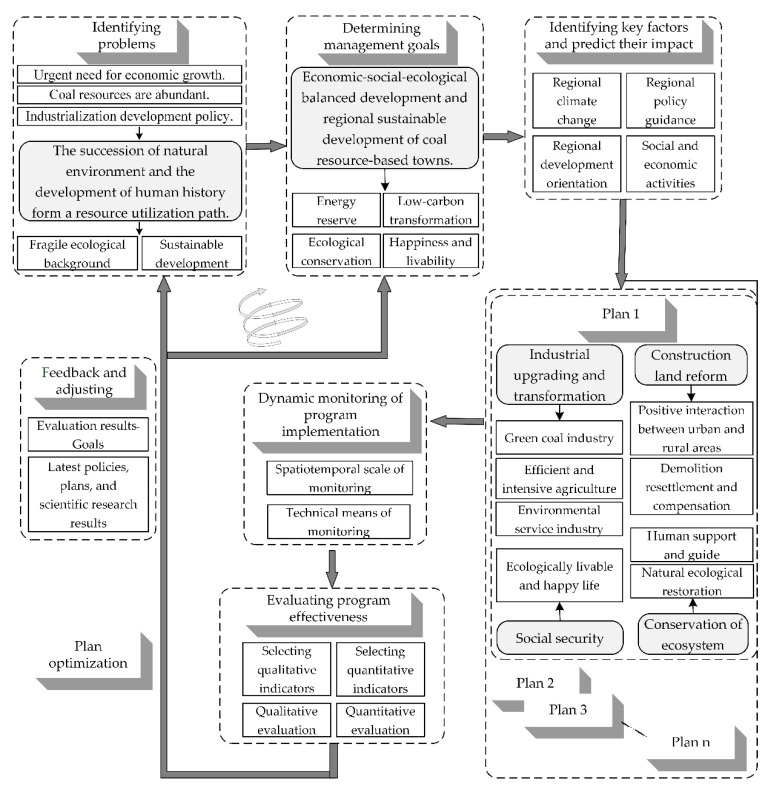
The framework of adaptive management of the social–ecological system of the mine–agriculture–urban compound area of Pinglu. (The logical framework of this figure refers to the research results of Wang et al. in 2007, Engle in 2011, Xia et al. in 2015, and Ma et al. in 2020. The content of this figure summarizes the text content in the above manuscripts, and the figure was made by Shufei Wang).

Figure 8 shows the logical framework of social–ecological system management in this compound area based on the above analysis and the determination of regional management objectives. The specific process includes: (1) Identifying problems in this area. Against the background of economic development needs, abundant resource reserves, and national industrialization development needs, the study area has formed a fragile ecological background and a weak development foundation, which is the key problem to be solved in the current regional sustainable development. (2) Determining management goals. In order to achieve sustainable development, the coordination and balance of economy, society, and ecology should be the future development goals in the social–ecological system of this compound area, including energy reserve, low-carbon transformation, ecological conservation, and happiness and livability. (3) Identifying key factors and predicting their impact, which are mainly carried out through climate change, policy guidance, regional development orientation, and social and economic activities dominated by mining and agriculture. According to different combinations of key factors, the corresponding future development scenarios can be simulated, and the key constraints in different scenarios can be identified. (4) Designing and implementing plans. Based on the constraints of each development scenario, corresponding management strategies can be designed, and a series of adaptive management plans can be formed. The adaptive management strategy should be proposed and implemented mainly through industrial upgrading and transformation, mining land reform, ecological maintenance and restoration, and grassroots employment and livelihood security. For example, ① by developing a green coal industry, efficient and intensive agriculture and environmental service industries, the upgrading and transformation of the industrial structure of the research area can be realized; ② By encouraging the positive interaction of urban and rural areas, and through scientific planning of the mining area demolition, resettlement and compensation policies to realize the goal of the construction land reform of the mining area; ③ By providing human support and guidance to assist the natural ecological restoration and achieve the goal of high-quality protection of the damaged ecological environment; ④ By building a social security system based on the goals of ecologically livable and happy life. (5) Dynamic monitoring of program implementation. The above plans should be monitored from multi-scale and multi-angle by using the remote sensing technique, low-altitude photographic surveying, and face-to-face interview surveys. (6) Evaluating program effectiveness. By reasonably selecting qualitative and quantitative indicators, and comparing the monitoring and analysis results with the set goals to evaluate the implementation process and results of each plan. (7) Optimizing the plan. Some good plans are selected based on the evaluation results. (8) Providing feedback and adjusting management processes based on the latest policies, plans, and scientific research results to update the problems identified originally, and to adjust the objectives and content of the preferred plans. In summary, through the continuous cycle of the adaptive management process, the optimal plan can finally be carried out to promote the sustainable and high-quality development of the social–ecological system in the mine–agriculture–urban compound area.

## 4. Discussion

### 4.1. Expansion of Adaptive Management in the Social–Ecological System Management of Typical Mine–Agriculture–Urban Compound Area

Based on the characteristic analysis, the social–ecological system of the mine–agriculture–urban compound area can be defined as: the complex ecological system formed under the disturbances of mining, agriculture, and urban construction, and under the influence of reclamation and restoration by artificial induction and natural resilience [12,46]. Compared with the general ecosystem, the social–ecological system of mine–agriculture–urban compound area emphasizes more the influence of human activities, and where the influence of human activities is strong, the ecological environment is seriously damaged and polluted, and the sustainable development of society, economy, and ecology faces great challenges [46,47].

**Figure 9:**
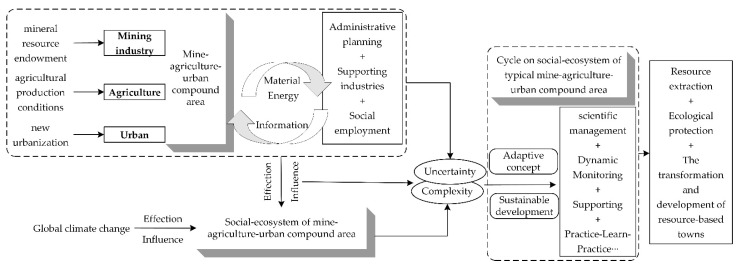
The theoretical framework of adaptive management of the social–ecological system of the mine–agriculture–urban compound area. (The logical framework of this figure refers to the research results of Salafsky et al. in 2001, Pelling and High in 2005, Yang et al. in 2014, and Ma et al. in 2020. The content of this figure summarizes the text content in the above manuscripts, and the figure was made by Shufei Wang).

Therefore, the adaptive management of the social–ecological system of mine–agriculture–urban compound area could be defined as: taking the social–ecological system of mine–agriculture–urban compound area as a whole; fully and dynamically understanding the structure and process of the ecological and social subsystems within the system; fully recognizing the disturbance and drive of changes in the external environment; considering the evolution of complexity, uncertainty, and limited predictability; being based on the theory of adaptability and regional coordination sustainable development; being based on scientific management, long-term monitoring, policy support, and public participation to identify the adaptive cycle process; continuously accumulating knowledge and experience; and forming a systematic, dynamic, gradual and cyclic upward plan to guide and regulate land use, resource development, and ecological restoration in the compound area in a timely manner, so as to reasonably promote the transformation and sustainable development of resource-based towns, to promote the balanced and coordinated development of social economy and ecological environment, and to ensure the integrity and sustainability of the structure and function of the ecosystem (Figure 9). Among these elements, the external disturbances and drivers, as well as the complexity, uncertainty, and limited predictability in the evolution, are emphasized for the resource exploitation and ecological restoration projects in the process of human mining, agriculture, and urbanization construction.

Through the analysis in “3.1. The development process” and “3.2. Characterization of social–ecological system”, we identified the problems existing in the study area and determined the goals of adaptive management corresponding to the current situation and demand analysis in the definition of adaptive management. The cycle-ascending adaptive management framework constructed in “3.3. The framework for adaptive management” is closely related to the definition of adaptive management. The investigation, design, planning, implementation, monitoring, evaluation, adjustment, and feedback in the definition of adaptive management are visually displayed through the management framework and process.

### 4.2. Applicability, Necessity, and Practical Value of Adaptive Management for Social–Ecological System of Typical Mine–Agriculture–Urban Compound Area

On the one hand, the social–ecological system of mine–agriculture–urban compound area is faced with many complex and uncertain factors originating from: (1) The internal structure and process of the ecosystem; (2) the destructive disturbance of mining, agriculture, and urbanization; (3) the governance of restorative disturbances in damaged ecosystems. Thus, the concept of adaptive management can be used to manage the social–ecological system of mine–agriculture–urban compound areas. On the other hand, the original ecosystem of the mine–agriculture–urban compound area indicated a certain change in law and circulation characteristics through its internal structure and process, and the disturbance effects of mining, agriculture, and urbanization on the original ecosystem obeyed certain social and economic rules, that is, a cyclical character tends to the next stable equilibrium state. That is, adaptive management should be applied to manage the social–ecological system of the mine–agriculture–urban compound area.

In addition, the adaptive management framework constructed in our study is applicable to the mine–agriculture–urban compound area or Pinglu District and has high a practical value. The *Territorial Spatial Planning of Shuozhou City (2020–2035)* released in 2021 positioned the future development of Pinglu as “ecological development”, and the core lay in the optimization of development methods and structures [39]. The *14th Five-Year Plan for National Economic and Social Development of Pinglu District of Shuozhou City and Outline of Vision for 2030* released in 2021 proposed that Pinglu would be economically prosperous in the future, residents would be harmonious and happy, and the ecological environment would be beautiful [48]. Among the proposals, economic prosperity depended on the green coal industry and efficient agriculture, residents’ harmony and happiness depended on economic growth and well-being, and a beautiful ecological environment depended on the green coal industry and protection and restoration planning [48]. The *Organization and Implementation Plan for the 2021–2022 Agricultural Production Trust Pilot Project in Pinglu District, Shuozhou City*, released in 2022, pointed out that the future development of this region should improve the level of mechanization, scale, and intensification of agricultural production, and should promote the formation of efficient and modern agricultural production [49]. The above-mentioned documents are the latest development planning documents of the Pinglu District. By summarizing the contents and comparing them with the contents of this research framework, it could be demonstrated that the management objectives and frameworks constructed in this study are in line with the practical needs of this region.

This research was a further development of the research on the mine–agriculture–urban compound area already carried out by Cao and Bai’s team. It could be seen from the summary that their research focused on the analysis of land-use change and ecosystem resilience in this region for about 30 years, and had little content about regional management [12,50]. In contrast, based on the analysis of the social and ecological system characteristics, this research focused on identifying the future development goals and management framework of the study area, which have a higher practical value and guiding role. That is, the novelty of this study lay in making clear and applicable recommendations based on sufficient research and theoretical foundations.

The adaptive management framework constructed in this study was aimed at the compound area formed by the close connection and roles of mining, agriculture, and urban development. The ideas and processes for the management framework construction could also be used as reference for all kinds of regional development, and many countries are currently facing the common needs of development security, ecological security, and food security, which require scientific development ideas and management frameworks. The management framework constructed in this study is focused on the co-development of mining, agriculture, and cities. So, when other regions apply this management framework, they can modify it according to regional development priorities.

### 4.3. Challenges of the Adaptive Management of the Social–Ecological System of Typical Mine–Agriculture–Urban Compound Area

The above-mentioned adaptive concepts and frameworks provide new and scientific ideas for compound area social–ecological system maintenance and restoration in complex and uncertain circumstances, but their current practice and realization process face additional problems and challenges.

(1) It is difficult to obtain timely information, make a reasonable judgment and offer feedback in the face of a constantly changing environment and policies as well as constantly updated knowledge and information, which is because human cognition and knowledge acquisition show lag and one-sidedness. A compound area social–ecological system is affected by natural, economic, and social factors, and managing it requires the knowledge of many disciplines such as ecology, restoration, management, systematics, and social sciences. However, the regions that mainly rely on the development of the coal mining industry need more knowledge of geology, engineering, and mechanical disciplines. That is, there is a lack of scholars and managers with comprehensive knowledge and literacy, as well as an insufficient understanding of the dynamic complexity of compound area social–ecological systems. These will affect identifying problems, determining management goals, identifying key factors, and predicting their impact in the adaptive management framework.

(2) In the stage of management goal determination and designing, implementing, and dynamic monitoring and evaluation of the program, it is difficult to select unified and widely recognized indicators that can be analyzed qualitatively and quantitatively. Qualitative and quantitative evaluation needs to establish an evaluation index system, but the evaluation parameters of different disciplines and different management objects are different. For example, economic and social evaluation is based on regional development indexes; ecosystem evaluation is based on landscape connectivity, vegetation coverage, and soil databases; and public perception evaluation is based on life satisfaction and happiness indexes. So, coordinating the contents of the management process and establishing an applicable evaluation index system are urgent problems to be solved.

(3) It is difficult to coordinate and balance the interests and demands of all stakeholders in the stage of management goal determination, formulation, and implementation of adaptive management programs. For example, the government needs regional development and good ecology, mining enterprises and financial institutions need economic benefits, scientific research institutes need local funds and venues support, and the public needs economic income and a beautiful environment. It is difficult to balance the interests of all parties and seek the optimal solution for regional development.

(4) It is difficult to deal with the time cost and provide stable financial support in the process of program formulation, implementation, evaluation, preference, and feedback. The management process takes a long time, during which the supervisor or the fund provider may change, which affects the sustainable progress of the management process.

(5) There are difficulties in achieving the full participation and recognition of grassroots people in the process of identifying problems, management strategy formulation, implementation, and dynamic monitoring. Adaptive management emphasizes the combination of participation with top-down and bottom-up management, and grassroots people are the ultimate beneficiaries of management results. Therefore, the management plans formulation requires extensive democratic discussion, and the implementation process requires the understanding and active participation of grassroots people. However, this is a missing link in current social development practice.

(6) It is difficult to persuade managers and grassroots people to change their fundamental development concept. Adaptive management is used in dealing with the uncertainty in development, and its implementation results also face a certain degree of uncertainty. Uncertainty regarding management results may cause local government managers to abandon management practices for political performance, or coal mining enterprises to deny the management in order to protect economic interests. At the same time, the grassroots people in coal mining areas have relied on coal mines for a long time to survive, their cultural level is generally low, their ability to acquire and use knowledge is limited, and it is more difficult to change their ideas than it is for other groups. All of these will affect the process of program formulation, implementation, monitoring, evaluation, preference, and feedback.

## 5. Conclusions

Firstly, this study combined the development history and policy documents to analyze the development trend of the mine–agriculture–urban compound area in detail, and identified its future development goals including energy storage, low-carbon transformation, ecological protection, and happiness and livability.

Secondly, combined with socio-economic statistics and land-use data, we analyzed the characteristics of the social–ecological system in the mine–agriculture–urban compound area and pointed out that the social and economic development of this area was mainly based on the coal mining industry, and the development potential of the agriculture industry could be fully utilized through the rational allocation of land resources. At the same time, because of the combined action of mining and agriculture, the regional ecosystem faced numerous instances of human disturbance and damage, and its governance was a necessary part of regional sustainable development requiring scientific and reasonable human guidance and management.

Finally, by summarizing the above content, we proposed the future development direction and management objectives of the mine–agriculture–urban compound area, which included solving the contradiction between development and protection, ensuring development, optimizing industrial structure, and protecting public interests. Among them, development is inevitable, solving the contradiction between development and protection is the core, optimizing the industrial structure is the key, and protecting the public interest is the foundation. At the same time, we built an adaptive management framework for the applicability and requirements of the region and indicated that although the application of the framework faces challenges, the framework is a management model that can be continuously improved through feedback and updated, and has strong advantages and potential.

This research can expand the application scope of the adaptive concept and provide a reference for similar areas facing severe development problems.

## Figures and Tables

**Table 1:** Summary of land use in the mine–agriculture–urban compound area. (The data were extracted from Figure 7, and the table was made by Shufei Wang).

Category	Subcategory	Percentage of the Area (%)
Mine	Opencast area Waste dump Striping area Industrial site	13.77
Agriculture	Cultivated land Forest land Grassland	81.71
Urban	Water area Urban land Rural settlement Transportation land	4.52

## Data Availability

Not applicable.
